# Identification and Validation of a Novel Pyroptosis-Related Gene Signature for Prognosis Prediction in Soft Tissue Sarcoma

**DOI:** 10.3389/fgene.2021.773373

**Published:** 2021-12-01

**Authors:** Lin Qi, Ruiling Xu, Lu Wan, Xiaolei Ren, WenChao Zhang, Keming Zhang, Chao Tu, Zhihong Li

**Affiliations:** ^1^ Department of Orthopaedics, The Second Xiangya Hospital, Central South University, Changsha, China; ^2^ Hunan Key Laboratory of Tumor Models and Individualized Medicine, The Second Xiangya Hospital, Changsha, China; ^3^ Department of Dermatology and Venereology, Changzheng Hospital, Second Military Medical University, Shanghai, China; ^4^ Shanghai Key Laboratory of Molecular Medical Mycology, Shanghai Institute of Medical Mycology, Changzheng Hospital, Second Military Medical University, Shanghai, China

**Keywords:** soft tissue sarcoma, pyroptosis, gene signature, prognosis, immune

## Abstract

Soft tissue sarcoma (STS) represents an uncommon and heterogenous group of malignancies, and poses substantial therapeutic challenges. Pyroptosis has been demonstrated to be related with tumor progression and prognosis. Nevertheless, no studies exist that delineated the role of pyroptosis-related genes (PRGs) in STS. In the present study, we comprehensively and systematically analyzed the gene expression profiles of PRGs in STS. The Cancer Genome Atlas (TCGA) and Genotype-Tissue Expression (GTEx) databases were utilized to identify differentially expressed PRGs. In total, 34 PRGs were aberrantly expressed between STS and normal tissues. Several PRGs were validated with RT-qPCR. Consensus clustering analysis based on PRGs was conducted to divide STS patients into two clusters, and significant survival difference was observed between two distinct clusters (*p* = 0.019). Differentially expressed genes (DEGs) were identified between pyroptosis-related clusters. Based on the least absolute shrinkage and selection operator (LASSO) COX regression analysis, the pyroptosis-related gene signature with five key DEGs was constructed. The high pyroptosis-related risk score group of TCGA cohort was characterized by poorer prognosis (*p* < 0.001), with immune infiltration and function significantly decreased. For external validation, STS patients from Gene Expression Omnibus (GEO) were grouped according to the same cut-off point. The survival difference between two risk groups of GEO cohort was also significant (*p* < 0.001). With the combination of clinical characteristics, pyroptosis-related risk score was identified to serve as an independent prognostic factor for STS patients. In conclusion, this study provided a comprehensive overview of PRGs in STS and the potential role in prognosis, which could be an important direction for future studies.

## Introduction

Soft tissue sarcomas (STSs) comprise a rare group of heterogenous tumor cells, which account for only 1% of all adult malignancies (Gamboa et al., 2020). It was estimated that there were 13,460 cases with STS in the United States in 2021 (Siegel et al., 2021). STS originated from mesenchymal tissues with more than 100 different subtypes according to the histology and genetic alterations, which displayed various clinical behaviors (Vilanova, 2017). Although STS could arise in any body site, it exhibited a predilection to occur in the extremity and intra-abdominal region (Brennan et al., 2014). Surgical procedures are the cornerstones of STS treatment (Crago and Brennan, 2015). The past few decades also have witnessed the evolution of therapeutic strategies for STS, with the collaboration of multidisciplinary team (MDT) including radiologists, pathologists, oncologists and surgical specialists (Gamboa et al., 2020). However, for elderly patients with STS, 5-years relative survival was below 50% (Hoven-Gondrie et al., 2016). Additionally, STS was also characterized by the susceptibility to distant metastasis and recurrence. Nearly half of patients with localized STS developed distant metastasis, especially to the lung and leading to poor prognosis (Navarria et al., 2015; Gamboa et al., 2020). Therefore, novel therapeutic targets and reliable prognostic model need to be identified for effective and personalized treatment.

Pyroptosis, recognized as caspase 1-dependent programmed cell death (PCD), features the prompt perforation of plasma membrane along with releasing intracellular properties of pro-inflammatory function (Bergsbaken et al., 2009; Shi et al., 2017). Pyroptosis is usually triggered by the activation of pattern recognition receptors (PRRs) and then activated caspase one upon inflammasomes (Xue et al., 2019). It was reported that pyroptosis-associated PRRs consist of intracellular nucleotide-binding oligomerization domain (NOD)-like receptors (NLRs), Toll-like receptors (TLRs) and absent in melanoma 2 (AIM2)-like receptors (ALRs) (Lamkanfi and Dixit, 2014). In recent years, gasdermin D (GSDMD) was reported as the executioner of pyroptosis, as it released N-terminal fragment (GSDMD-cNT) to induce cell swelling after caspase cleavage (Shi et al., 2015; Ding et al., 2016). Likely, other gasdermin family genes including GSDMA, GSDMB, GSDMC and GSDME also take part in the process of pyroptosis (Ding et al., 2016). In the pathophysiologic process of diseases, pyroptosis is competitively regulated between the host and pathogen, and the outcomes determine the fate of the host in turn (Bergsbaken et al., 2009).

The tight relationship between pyroptosis and cancers has been reported in recent years, while controversy still exists regarding the dual role of pyroptosis (Xia et al., 2019). Various signaling pathways activated by pyroptosis may promote tumorigenesis and chemoresistance (Thi and Hong, 2017; Zhou and Fang, 2019). Nevertheless, pyroptosis may also exert tumor-suppressive effect by inhibiting tumor growth and angiogenesis (Nagarajan et al., 2019). In hepatocellular carcinoma (HCC), significant downregulation of NLR family pyrin domain containing 3 (NLRP3) was observed, which was inversely correlated with clinical stage (Wei et al., 2014). Highly expressed GSDMB was associated with poor prognosis and high metastatic potential in breast cancer (Hergueta-Redondo et al., 2014). Furthermore, there has been no relevant study focusing on the role of pyroptosis in STS. With genomic and clinical information integrated, the current study aims to systematically analyze pyroptosis-related genes (PRGs) in STS, develop and validate pyroptosis-related risk score and establish the novel prognostic model for STS.

## Materials and Methods

### Data Collection and Sources

The UCSC Xena browser (https://xenabrowser.net/datapages/) was used to download gene expression profiles of The Cancer Genome Atlas (TCGA)—sarcoma (SARC) cohort and normal tissues in the Genotype-Tissue Expression (GTEx) dataset (Goldman et al., 2020). FPKM values of RNA sequencing (RNA-Seq) data from TCGA and GTEx were normalized through log_2_(FPKM+1) transformation. RNA-Seq data from two database were then processed and unified following sufficiently rigorous procedures, including the uniform realignment, the quantification of gene expression and the correction of batch effect (Wang et al., 2018). Clinical information of TCGA-SARC cohort has been made available for download at cBioPortal (https://www.cbioportal.org/) (Cerami et al., 2012). Within TCGA-SARC cohort, a total of 259 patients with STS were screened, composed of 104 patients having leiomyosarcoma (LMS), 59 patients having dedifferentiated liposarcoma (DDLPS), 49 patients having undifferentiated pleomorphic sarcoma (UPS), 25 patients having myxofibrosarcoma (MFS) and 22 patients having other STS. In GTEx, gene expression profiles of 911 normal human adipose and muscle were integrated with that of TCGA-cohort owing to the lack of normal tissues in TCGA. Additionally, the RNA-Seq profiles with clinical characteristics of GSE30929 were available in the GEO data repository for further validation.

### Identification of Differentially Expressed genes Between Pyroptosis-Related Clusters

In total, 37 PRGs were identified based on previous studies (Kang et al., 2014; Zhu et al., 2017; Feng et al., 2018; Tsuchiya et al., 2019; Xue et al., 2019; Zhang et al., 2020; Zheng and Kanneganti, 2020), and were listed in Supplementary Table S1. Differential expression of PRGs between SARC and corresponding normal tissue was conducted, utilizing the “limma” R package (Version 3.48.3). In clusters based on consensus clustering analysis of PRGs, DEGs between cluster one and cluster two were identified if | log_2_ (fold change) | > 2 in expression value and false discovery rate (FDR) < 0.05. We established correlation network of PRGs using “corr” R package (Version 0.4.3) with the correlation coefficient set of 0.4. Somatic mutation of PRGs were visualized utilizing “Maftools” R package (Version 2.8.0). Circos plot was present to demonstrate the location of PRGs by “Circos” R package (Version 1.2.1).

### Protein–Protein Interaction Network for PRGs

The PPI network of PRGs was constructed with the minimum required interaction score set of 0.9 to ensure high confidence, utilizing STRING database (https://string-db.org/) (Szklarczyk et al., 2021). Moreover, PPI network of PRGs was further analyzed using Cytoscape software (version 3.8.2). Hub genes were then screened based on MCODE, with the indicators set as degree cutoff = 2, node score cutoff = 0.2, K-core = 2 and maximum depth = 100.

### Pyroptosis-Based Consensus Clustering Analysis

The “ConsensusClusterPlus” R package (Version 1.56.0) was introduced to conduct consensus clustering analysis, so as to identify pyroptosis-related subtypes of STS. Because k-means clustering analysis was stochastic, repetitions was set to 1,000 to ensure stable clustering (Wilkerson and Hayes, 2010). Differences in survival among clusters were visualized with Kaplan-Meier (KM) plots based on the R packages of “survival” (Version 3.2–11) with “survminer” (Version 0.4.9).

### Gene Set Enrichment Analysis

DEGs (| log_2_ (fold change) | > 2 in expression value and FDR <0.05) between cluster one and cluster two based on pyroptosis-related consensus clustering were collected. With the “clusterProfiler” R package (Version 4.0.4) introduced, Gene Ontology (GO) and Kyoto Encyclopedia of Genes and Genomes (KEGG) enrichment analysis were performed.

### Development of Pyroptosis-Based Prognostic Model

The prognostic significance of DEGs within different clusters was evaluated by utilizing univariate COX regression analysis. In order to narrow down gene selection, DEGs with significant impact on survival (*p* < 0.01) were subsequently incorporated into the least absolute shrinkage and selection operator (LASSO) Cox regression analysis based on the “glmnet” R package (Version 4.1–2). The risk scores were further established according to the formula: $\overset{n}{\underset{i}{\sum }}Xi*Yi$ (*Xi*: coefficients of the gene *i*, *Yi*: expression values of the gene *i*).

The risk scores were subsequently discretized to divide TCGA-SARC cohort into high- and low-risk groups. Survival between two risk groups was analyzed with KM plot in TCGA-SARC cohort. For further external validation of risk scores, gene expression profiles of GSE30929 were entered into the formula, and we sorted these patients into high- and low-risk groups according to the same cut-off point. The “timeROC” R package (Version 0.4) was introduced for evaluating the predictive accuracy. Furthermore, the risk scores integrated with clinical characteristics including age, sex, race, histology, tumor site, tumor multifocality, surgical margin, tumor depth and radiotherapy were analyzed using multivariate COX regression analysis. The nomogram was performed to illustrate the prognostic model, which was further evaluated by the calibration curve.

### Single Sample Gene Set Enrichment Analysis and Immune Infiltration Analysis

The 16 immune cells infiltration and 13 related functions were quantified through ssGSEA in different pyroptosis-related risk groups of TCGA-SARC cohort and GSE30929, by utilizing the R package of “GSVA” (Version 1.40.1) and “GSEABase” (Version 1.54.0). The correlation between expression of DEGs in the gene signature and immune infiltrates was analyzed by the Tumor Immune Estimation Resource 2.0 database (TIMER2.0) (Li et al., 2020).

### Cell Lines and Cell Culture

The human synovial sarcoma cell line (SW-982) was purchased from the American Type Culture Collection (ATCC). The human skin fibroblast cell line (HSF) with related media were purchased from Fenghui Biotechnology Co., Ltd (Hunan, China). The primary human synovial sarcoma cells (hSS-005R) were also established for the validation of PRGs. The human synovial sarcoma cells SW-982 and hSS-005R were cultured in Dulbecco’s modified Eagle medium (DMEM) (Gibco, United States) supplemented with 10% fetal bovine serum (FBS) (Gibco, United States) and 1% penicillin-streptomycin (NCM Biotech, China). Cells were cultured at 37°C with 5% CO_2_ in a humidified incubator (Thermo Fisher Scientific, United States).

### Real-Time Quantitative PCR

Total cellular RNA was extracted using the RNA Express Total RNA Kit (M050, NCM Biotech, China). For cDNA synthesis, reverse transcription was conducted with the RevertAid First Strand cDNA Synthesis kit (K1622, Thermo Fisher Scientific, United States). Subsequently, RT-qPCR was performed on the StepOne Plus (Applied Biosystems, United States) by utilizing SYBR Green qPCR Master Mix (2×) (Bimake, United States). The primers used for the RT-qPCR were listed in Table 1.

**TABLE 1:** Sequences of the primers used in RT-qPCR.

Gene	Sequence of primer
*CASP3*	F: CAT​GGA​AGC​GAA​TCA​ATG​GAC​T
	R: CTG​TAC​CAG​ACC​GAG​ATG​TCA
*DHX9*	F: GCA​GCA​GAG​TGT​AAC​ATC​GTA​G
	R: ACT​CAA​ATC​GAA​CGC​TGT​AGC
*IL1B*	F: ATG​ATG​GCT​TAT​TAC​AGT​GGC​AA
	R: GTC​GGA​GAT​TCG​TAG​CTG​GA
*GADPH*	F: CAG​GAG​GCA​TTG​CTG​ATG​AT
	R: GAAGGCTGGGGCTCATTT

UPS, undifferentiated pleomorphic sarcoma.

### Statistical Analysis

Statistical Analysis were conducted by utilizing R (Version 4.1.0). Differential gene expression between two groups was identified using Wilcoxon rank sum test, with *p* value calculated for each gene. Spearman’s correlation test and matrix were conducted to compare gene expression with each other. Survival differences were compared utilizing log-rank test with KM curve. The χ2 test or Fisher’s exact test was introduced to evaluate clinical characteristics between high- and low-risk groups. COX regression analysis was conducted to identify prognostic factors, and hazard ratio (HR) with 95% confidence interval (CI) were also computed. Statistical difference of *p* < 0.05 was defined significant.

## Results

### Identification of PRGs Between STS and Normal Tissues

**FIGURE 1:**
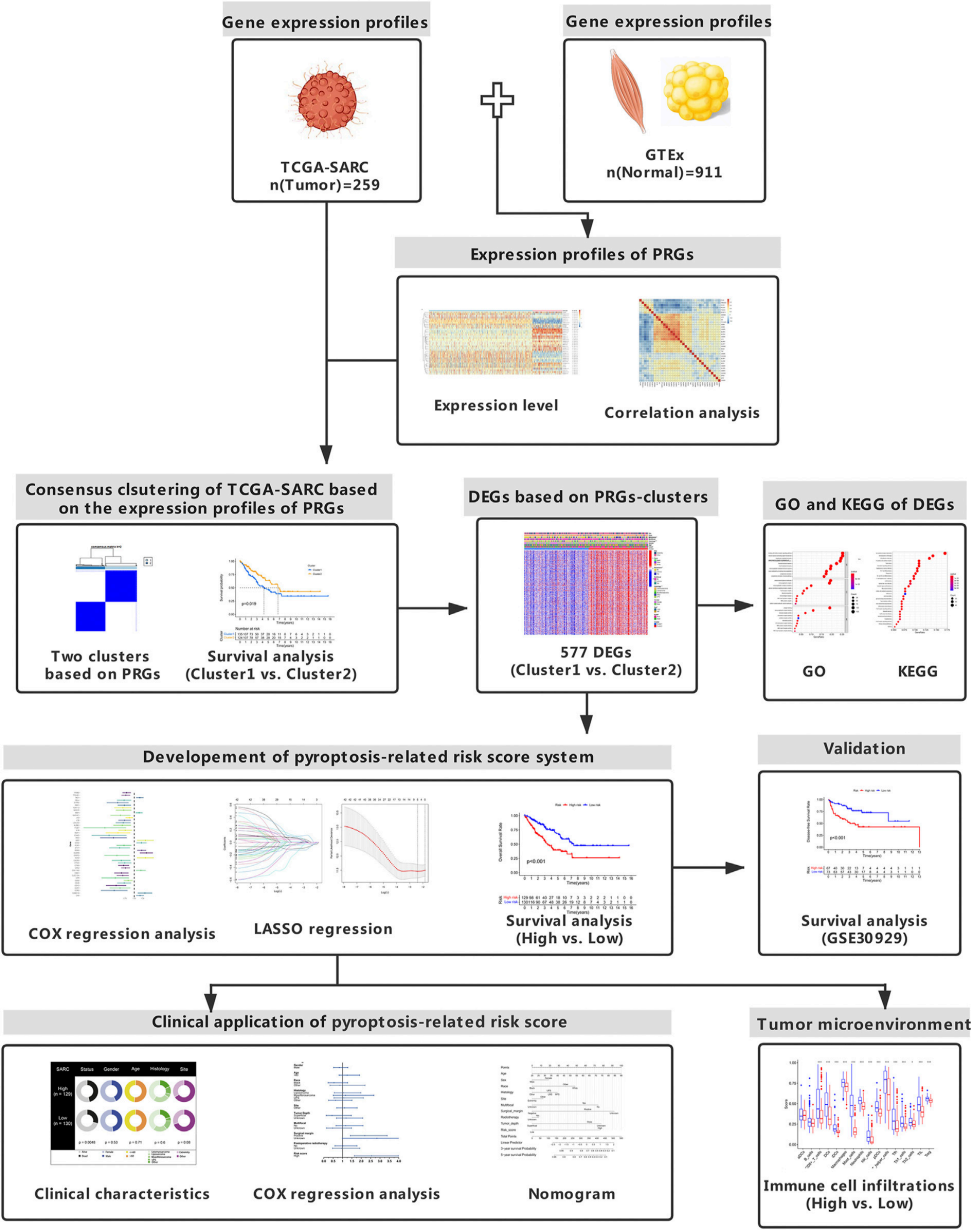
Study design. The flowchart presents the process of data collection and analysis.

**FIGURE 2:**
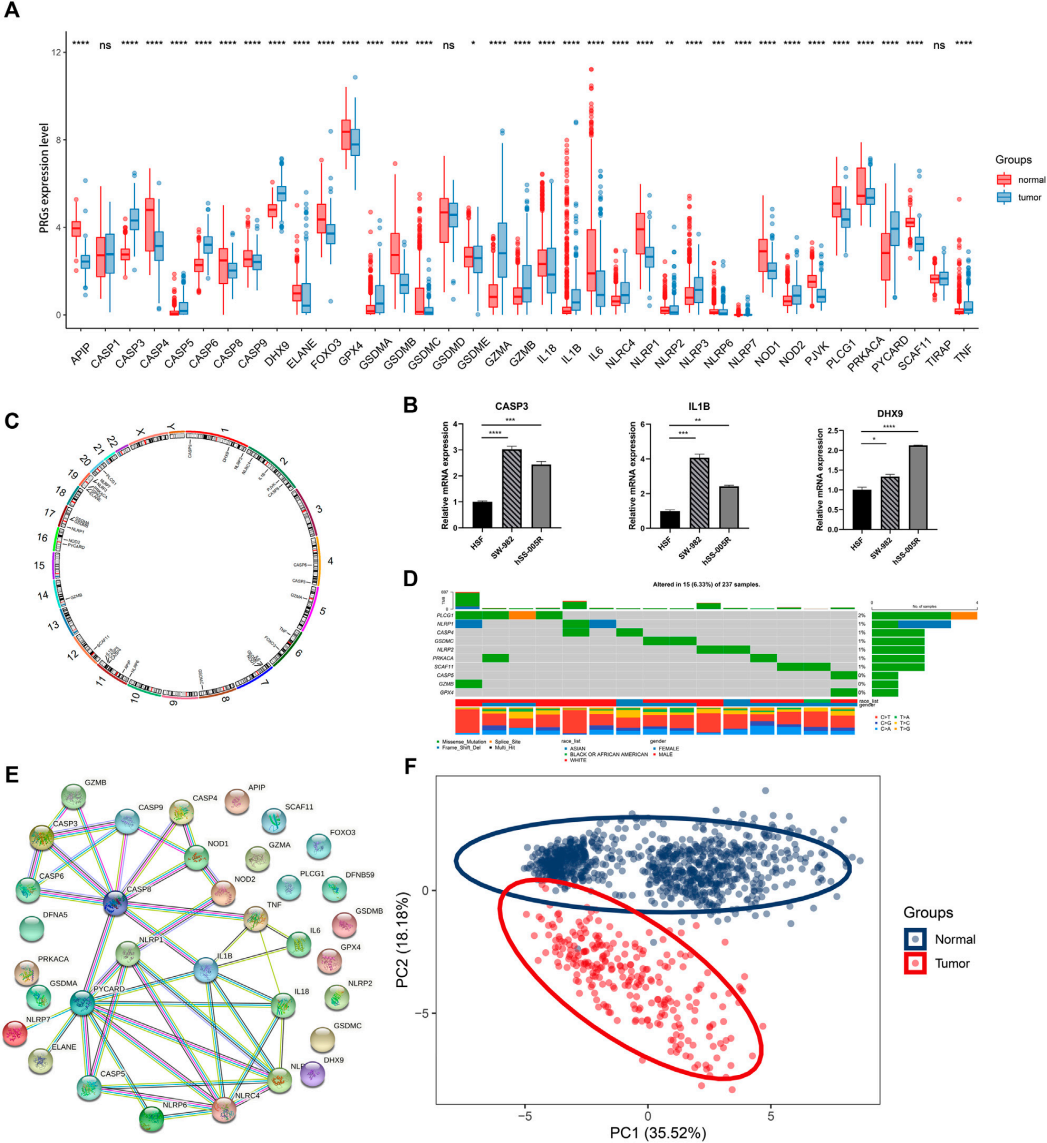
Expression and association of PRGs. **(A)** The expression of PRGs between STS (blue) and normal tissues (red). Box plot represents the median (center horizontal line), upper and lower quartiles (top and bottom horizontal line). **(B)** Validation of mRNA expression of key PRGs in cell lines. **(C)** The location of PRGs on chromosomes. **(D)** Schematic overview of mutation frequency and type in PRGs. **(E)** PPI network constructed by PRGs-encoded proteins (interaction score: 0.9). **(F)** Principal component analysis (PCA) for discriminating STS and normal tissues based on PRGs. *p < 0.05, **p < 0.01, ***p < 0.001, ****p < 0.0001, ns: nonsignificant.

The study design was illustrated in Figure 1. Totally 37 PRGs were included to detect differential expression between STS and normal tissues from TCGA-SARC and GTEx dataset (Supplementary Table S1). We found that 34 PRGs were differentially expressed (*p* < 0.05), among which 14 genes (CASP3, CASP5, CASP6, DHX9, GSDMA, GZMA, GZMB, IL1B, NLRC4, NLRP3, NLRP7, NOD2, PYCARD, TNF) were upregulated and 20 genes (APIP, CASP4, CASP8, CASP9, ELANE, FOXO3, GPX4, GSDMB, GSDMC, GSDME, IL18, IL6, NLRP1, NLRP2, NLRP6, NOD1, PJVK, PLCG1, PRKACA, SCAF11) were downregulated in STS group (Figure 2A, Supplementary Figure S1A). To validate PRGs in related cell lines, we performed RT-qPCR analysis (Figure 2B). The expression levels of several key PRGs including CASP3, IL1B and DHX9 were significantly higher in the human synovial sarcoma cells SW-982 and hSS-005R, compared with those in the human skin fibroblast cell line (HSF). The correlation network of 34 PRGs was present in Supplementary Figure S1B and Supplementary Figure S1C. The chromosome location of 34 PRGs was illustrated in Figure 2C. We then analyzed copy number variations (CNVs) of PRGs (Figure 2D). It could be found that 15 of 237 (6.33%) SARC samples displayed pyroptosis-related mutations. And the majority of mutations were missense mutations. The PPI network was subsequently established and seven hub genes (NLRP1, IL18, NLRC4, NLRP3, PYCARD, CASP5, IL1B) were identified (Figure 2E). Furthermore, these 34 PRGs were efficient to discriminate STS and normal tissues on the expression level (Figure 2F).

### Identification of TCGA-SARC Cluster Based on PRGs

**FIGURE 3:**
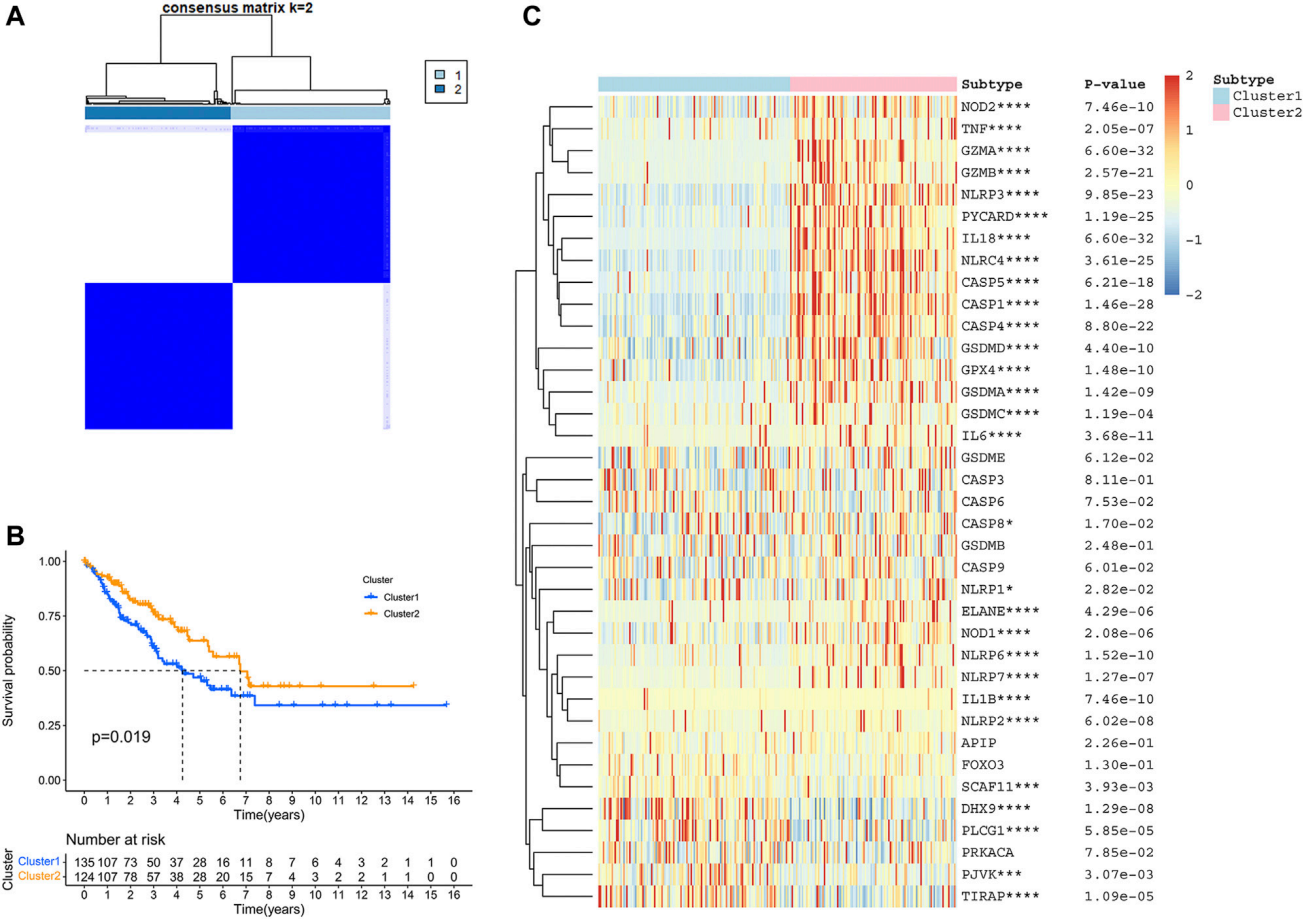
Identification of TCGA-SARC cluster based on PRGs. **(A)** TCGA-SARC cohort was divided into two distinct clusters using pyroptosis-based consensus clustering analysis (k = 2, repetition = 1,000). **(B)** Overall survival (OS) curve comparing survival of patients in cluster 1 (blue) and cluster 2 (orange). **(C)** Heatmap of PRGs between two clusters (*p < 0.05, **p < 0.01, ***p < 0.001, ****p < 0.0001).

To elucidate different STS subtypes and corresponding clinical characteristics and prognosis, the TCGA-SARC cohort was clustered into two distinct clusters based on PRGs through consensus clustering analysis (Figure 3A, Supplementary Figure S2A–F). Within pyroptosis-related cluster 1, There were 135 STS patients and 124 STS patients were in pyroptosis-related cluster 2. Remarkably, overall survival (OS) curves of these two clusters indicated significantly great survivorship difference (*p* = 0.019, Figure 3B). In Figure 3C, the gene expressing level of PRGs in two distinct clusters were displayed.

### Profiling DEGs Between Pyroptosis-Related Clusters

**FIGURE 4:**
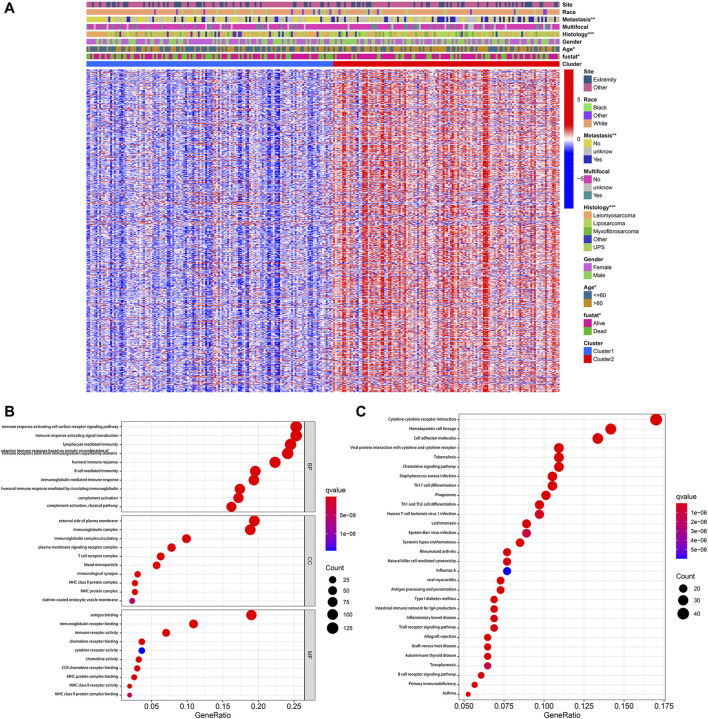
Identification of DEGs between pyroptosis-related clusters. **(A)** Heatmap depicting DEGs of pyroptosis-related clusters and corresponding clinical characteristics. **(B)** GO enrichment analysis including biological process (BP), cellular component (CC), and molecular function (MF). **(C)** KEGG enrichment analysis indicating related genes and pathways. *p < 0.05, **p < 0.01, ***p < 0.001.

According to the stringent selecting criterion of | log_2_ (fold change) | > 2 in expression value and FDR <0.05, a total of 577 DEGs were identified between pyroptosis-related cluster one and cluster 2 (Figure 4A). There were also significant differences in clinicopathological characteristics including age, histology, metastatic status and survival between two distinct clusters (*p* < 0.05).

Subsequently, these 577 DEGs were subjected to the GO and KEGG enrichment analysis, in order to reveal biological processes and mechanisms of pyroptosis-related clusters. GO enrichment analysis indicated that DEGs were predominantly enriched in immune response-activating cell surface receptor signaling pathway, immune response-activating signal transduction, external side of plasma membrane and antigen binding (Figure 4B). Moreover, these DEGs were also significantly enriched in cytokine-cytokine receptor interaction, hematopoietic cell lineage and cell adhesion molecules (Figure 4C).

### Development and Validation of Pyroptosis-Related Gene Signature in STS

**FIGURE 5:**
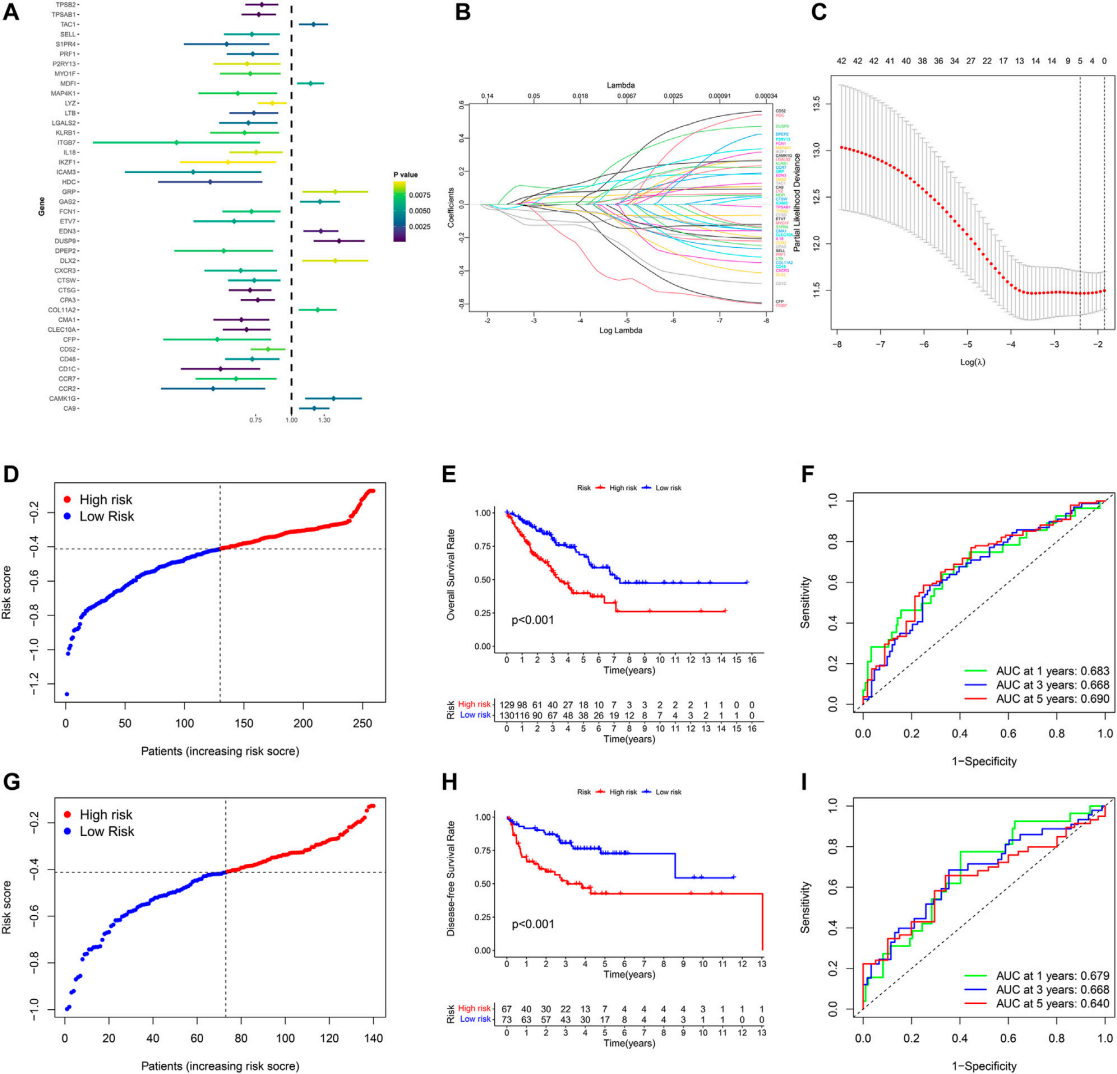
Development and validation of the pyroptosis-related gene signature in STS. **(A)** Univariate COX regression analysis of DEGs between pyroptosis-related cluster one and cluster 2, and all 42 genes with *p* < 0.001. **(B)** LASSO regression analysis of 42 DEGs. **(C)** Cross validation method to select optimal genes. **(D)** Distribution of TCGA-SARC cohort based on the risk score. **(E)** OS curve of TCGA-SARC cohort in low-risk and high-risk groups. **(F)** Time-dependent ROC to evaluate the prognostic performance of the risk score in TCGA-cohort. **G** Distribution of GSE30929 cohort based on the risk score. **(H)** Disease-free survival (DFS) curve of GSE30929 cohort in low-risk and high-risk groups. **(I)** Time-dependent ROC to evaluate the prognostic performance of risk score in GSE30929 cohort.

The prognostic significance of 577 DEGs between cluster one and cluster two were analyzed by utilizing univariate COX regression analysis. Accordingly, 42 genes were preserved based on the strict criteria (*p* < 0.01) and processed for subsequent analysis (Figure 5A). The LASSO COX regression analysis was then performed, and five key genes were eventually identified with the pyroptosis-related gene signature constructed (Figures 5B,C). The risk score = (-0.05167*CTSG exp.) + (0.06184*DUSP9 exp.) + (-0.02483*CLEC10A exp.) + (-0.02979*CPA3 exp.) + (-0.06014*CD1C exp.). Based on the median of the risk scores in TCGA-SARC cohort, 259 STS patients were divided into the low-risk group (n = 130) and the high-risk group (n = 129) (Figure 5D, Supplementary Figure S3A). Principal component analysis (PCA) and t-distributed stochastic neighbor embedding (t-SNE) indicated that low-risk and high-risk group could be clearly distinguished (Supplementary Figure S3C, Supplementary Figure S3E). The KM plot of OS rate demonstrated significant difference between two risk scores groups of TCGA-SARC cohort (*p* < 0.001, Figure 5E). Time-dependent receiver operating characteristics (ROC) curves were introduced for assessing model performance, and the area under curve (AUC) of 1-year, 3-year and 5-year OS rate were 0.683, 0.668 and 0.690, accordingly (Figure 5F).

For further validating the accuracy of the pyroptosis-related gene signature, corresponding data of GSE30929 was retrieved and the risk score of was calculated respectively. According to the same cut-off point of TCGA-SARC cohort, GSE30929 cohort was divided into different risk groups (Figure 5G, Supplementary Figure S3B). PCA and t-SNE also illustrated optimal degree of discrimination between high- and low-risk groups of GSE30929 (Supplementary Figure S3D, Supplementary Figure S3F). Remarkably, the disease-free survival (DFS) of two distinct risk groups demonstrated significant discrepancy (*p* < 0.001, Figure 5H). AUC were 0.679 for 1-year, 0.668 for 3-year and 0.640 for 5-year (Figure 5I).

### Development of Pyroptosis-Based Prognostic Model

**FIGURE 6:**
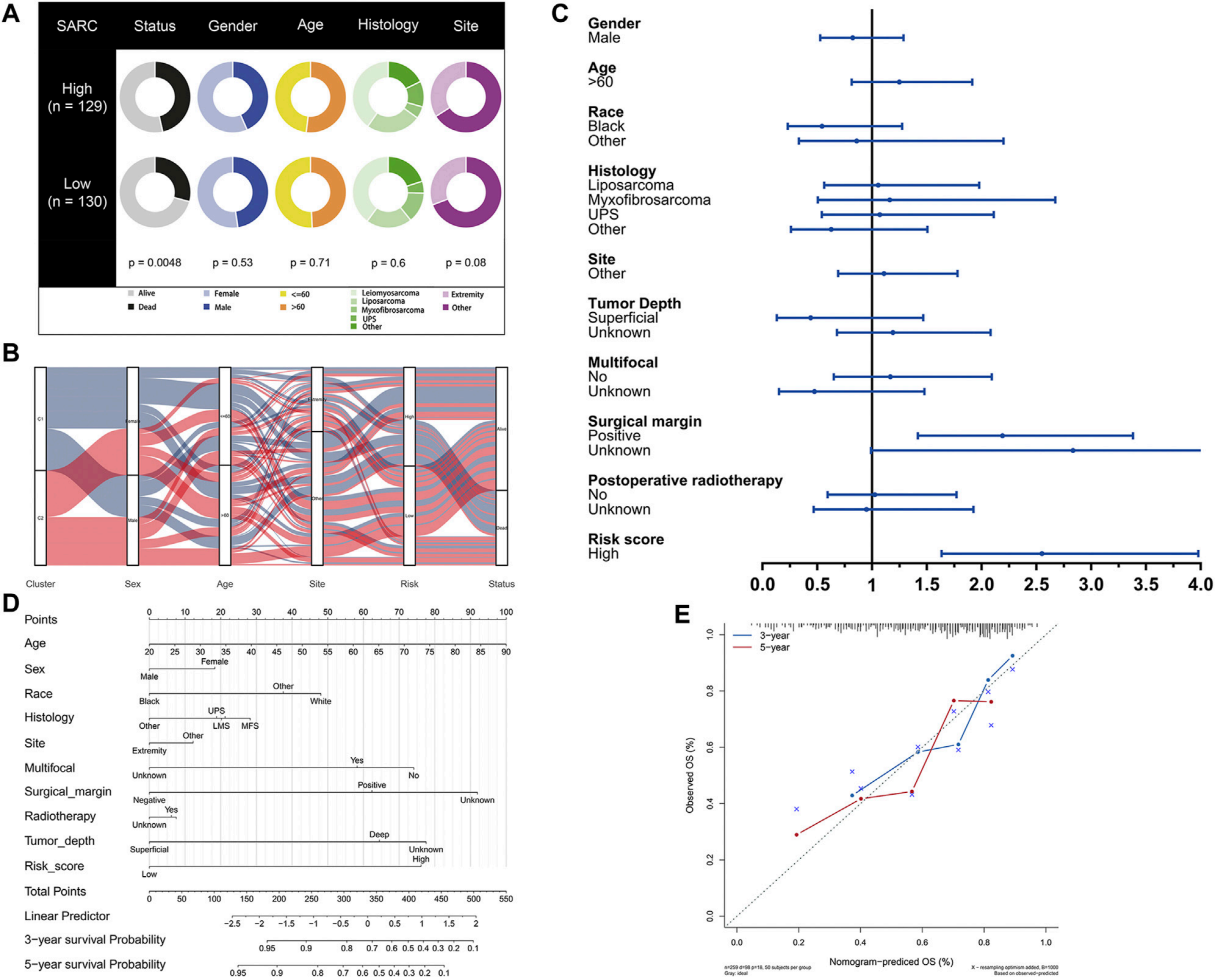
Development of pyroptosis-based prognostic model. **(A)** Clinical characteristic between low-risk and high-risk groups. **(B)** Alluvial diagram illustrating the relationship of pyroptosis-based cluster distribution, clinical characteristics, different risk groups and survival outcomes. **(C)** Multivariate COX regression analysis of clinical characteristics and pyroptosis-based risk score. **(D)** Nomogram predicting 3-years and 5-years survival rate of STS patients. **(E)** Calibration curve for predicting OS rate of STS patients.

The potential clinical utility of pyroptosis-based risk score was further investigated. Clinical characteristics of gender, age, tumor histology and tumor site between high- and low-risk groups were visualized in Figure 6A. Alluvial diagram also illustrated the relationship of pyroptosis-based cluster distribution, clinical characteristics, different risk groups and survival outcomes (Figure 6B). Furthermore, multivariate COX regression analysis integrated with clinical characteristics and pyroptosis-based risk score were performed to establish the prognostic model (Figure 6C, Supplementary Table S2). Based on the established prognostic model, a novel nomogram was subsequently constructed for predicting the survival probability of STS patients (Figure 6D). The 3-year and 5-year OS rate have proven to be relatively well predicted by the calibration curve of the nomogram (Figure 6D).

### Analysis of Immune Status Based on Pyroptosis-Related Risk Score

**FIGURE 7:**
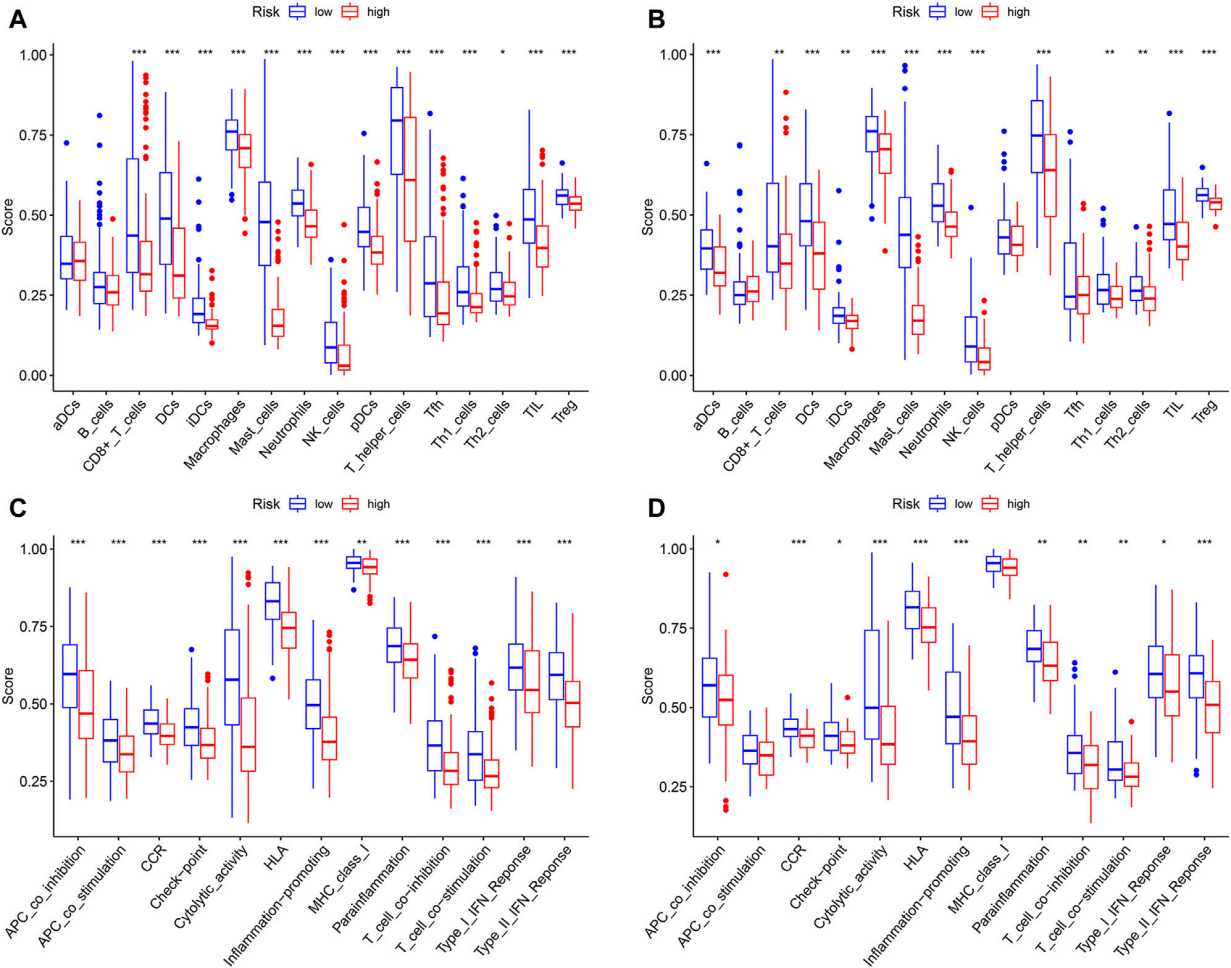
Analysis of immune status based on pyroptosis-related risk score. **(A, C)** Comparisons of immune cells and immune functions between different risk groups in TCGA-SARC cohort. **(B, D)** Comparisons of immune cells and immune functions between different risk groups in GSE30929 cohort (*p < 0.05, **p < 0.01, ***p < 0.001).

In order to compare the immune activity, the ssGSEA was applied to analyze the immune infiltration and functions of high- and low-risk groups. The ssGSEA score calculated could indicate the infiltration degrees of immune cells and pathways within TCGA-SARC cohort and GSE30929 cohort. In TCGA-SARC cohort, the infiltration degrees of CD8^+^ T cell, dendritic cell (DC), immature DC (iDC), macrophage, mast cell, neutrophil, natural killer (NK) cell, plasmacytoid DC (pDC), T helper (Th) cell, T follicular helper (Tfh) cell, Th1 cell, Th2 cell, tumour-infiltrating lymphocyte (TIL) and regulatory T cell (Treg) were significantly lower in the high pyroptosis-related risk group (*p* < 0.05, Figure 7A). All 13 related immune functions were also significantly decreased in the high pyroptosis-related risk group (*p* < 0.05, Figure 7C). GSE30929 dataset was subsequently included to analyze the immune activity. The results were similar with the majority of immune infiltration and function significantly decreased in the high-risk group (Figure 7B, Figure 7D). The correlation of key DEGs in the gene signature and immune infiltration was also illustrated (Supplementary Figure S4A–E).

## Discussion

For a long period of time, apoptosis has been traditionally considered as the predominant mode that regulating cell death (Lowe and Lin, 2000; Reed, 2000). With further studies on novel forms of cell death, pyroptosis has aroused increasing attention due to its morphological and mechanistical distinction from others (Bergsbaken et al., 2009). Besides, pyroptosis was reported to chemically mediate multiple processes of malignancy progression (Wang et al., 2019; Ruan et al., 2020). However, no study has yet investigated the role of pyroptosis in STS. In this study, we comprehensively analyzed gene expressing profiles of PRGs in STS. Moreover, pyroptosis-related risk scoring system was established and validated.

The gene expression of 34 in 37 predefined PRGs was found to be significantly different between STS and normal tissues in the current study. Similarly, PRGs included in this study were relatively consistent with those in studies focusing on pyroptosis in other tumor types (Lin et al., 2021; Ye et al., 2021). Due to constraints of the TCGA data, normal tissue samples were extremely limited in TCGA-SARC cohort. Thus, GTEx including expression profiles of normal human tissues was the optimal data resource (Carithers et al., 2015). The gene expression data of TCGA and GTEx were merged through sufficiently rigorous procedures (Wang et al., 2018), and this novel method has been confirmed by several studies concerning TCGA-SARC cohort (Hu et al., 2020; Huang et al., 2021). Besides, PRGs identified in this study showed good ability to distinguish STS from normal samples. Based on the differentially expressed PRGs sets, tumor-infiltrated and normal tissues could be clearly distinguished, which was strongly suggestive of the role in tumor diagnosis. Consensus clustering analysis was a proven method to demonstrate distinct subtypes and survival patterns of malignant tumors (Brannon et al., 2010; Wang et al., 2014). The clustering of subtypes of TCGA-SACR cohort provided an opportunity to identify biological differences of STS based on PRGs. STS patients in pyroptosis-related cluster two had substantially better prognosis, with most PRGs significantly upregulated within this cluster. Remarkably, the previous study has demonstrated that the ATP releasing by dying tumor cells would act on P2X_7_ purinergic receptors and subsequently trigger NLRP3-CASP1 complex (inflammasome) to mediate the innate and adaptive immune responses against dying tumor cells (Ghiringhelli et al., 2009). In keeping with previous findings, the current study also identified significant upregulation of NLRP3 and CASP1 in the pyroptosis-related cluster 2, which was probably related with underlying mechanisms of different prognosis in STS clusters.

The DEGs analysis between two pyroptosis-related clusters was further conducted to establish the risk score system, which was also a proven method of identifying different risk tumor patterns (Bai et al., 2021). Gene enrichment analysis revealed the significant enrichment in several immune-related pathways, and these findings were also coincident with the role of pyroptosis in mediating immune system (Hachim et al., 2020). Importantly, pyroptosis-related signature was established based on five key DEGs by LASSO COX regression analysis. Besides, solely relying on a single gene for diagnosis and prognosis prediction was inaccurate (Ju et al., 2021). The utility of pyroptosis-related risk score was confirmed by the significant survival difference in TCGA-SARC cohort. As relevant STS dataset in Gene Expression Omnibus (GEO) was extremely limited, there was no OS status recorded in GSE30929, and only DFS status were available. However, to our surprise, this pyroptosis-related risk score was also significantly efficient to predict the DFS of GSE30929 cohort for external validation. There were studies demonstrating that DFS could be considered as the acceptable surrogate of OS in a variety of tumors (Fajkovic et al., 2013; Oba et al., 2013), which hinted potential relationship between OS and DTS in STS.

After adjusting for clinical characteristics, pyroptosis-related risk score turned out to be the independent prognostic factor for overall survival of TCGA-SARC cohort. Besides, the nomogram integrated with pyroptosis-related risk score and clinical indicators has been developed for the clinical application. The score of each indicator could be added to estimate the OS rate of patients with STS. In the high pyroptosis-related risk group, the immune infiltration degrees were significantly lower, also indicating abnormal immune functions (Pages et al., 2010). Dual-specificity phosphatase 9 (DUSP9), one gene of the pyroptosis-related signature, is a dual-specificity phosphatase inhibiting mitogen-activated protein kinases (MAPKs) with preference for ERK (Caunt and Keyse, 2013). Multiple studies have revealed the relationship between DUSP9 and different types of tumors (Lu et al., 2018; Chen et al., 2021). In the current study, the coefficient of DUSP9 was positive in the formula of the pyroptosis-related risk score, which contributed the most to the increasing of the risk score and may suggest several directions towards relevant fields.

To our best knowledge, this is the first study identifying pyroptosis-related gene signature in STS, which is of great significance in diagnosis and survival prediction. However, the sample size of STS was limited due to disease characteristics, which was one of the deficiencies of this study. K-means clustering performed with k = 2 was also based on the limited sample size. Besides, most clinical data were collected retrospectively, and several important clinical characteristics including tumor grade, tumor stage, and surgy information were not available, which leading to some inevitable bias. Therefore, findings in this study should be viewed as the resource for future studies.

In conclusion, this study comprehensively and systematically analyzed the gene expression profiles of PRGs in STS. A total of 34 differentially expressed PRGs were identified, which were efficient to discriminate STS and normal tissues. Distinct pyroptosis-related clusters were divided with corresponding DEGs analyzed. Furthermore, pyroptosis-related risk scoring system with five key DEGs was established and served as an independent prognostic factor for STS patients. There was a significant difference in the levels of immune infiltration between low and high pyroptosis-related risk groups.

## Data Availability

Publicly available datasets were analyzed in this study. This data can be found here: TCGA-SARC and GSE30929.

## Supplementary Material

Supplementary Figure S1 Expression and association of PRGs. A The heatmap of PRGs between STS and normal tissues (*p < 0.05, **p < 0.01, ***p < 0.001, ****p < 0.0001). B and C The correlation network of PRGs.

Supplementary Figure S2 Consensus clustering of TCGA-SARC cohort based on PRGs. A-D Consensus clustering based on PRGs (k = 3-6). E Consensus cumulative distribution function (CDF) Plot based on PRGs. F Delta area plot of consensus clustering based on PRGs.

Supplementary Figure S3 Identification of low-risk and high-risk group based on risk score. A The survival status of TCGA-SARC cohort based on the risk score (low-risk group: left-hand side of vertical dotted line, high-risk group: right-hand side of vertical dotted line). B The survival status of GSE30929 cohort based on the risk score. C PCA for TCGA-SARC cohort based on the risk score. D PCA for GSE30929 cohort based on the risk score. E t-SNE for TCGA-SARC cohort based on the risk score. F t-SNE for GSE30929 cohort based on the risk score.

Supplementary Figure S4 The correlation of prognostic DEGs of the gene signature and immune infiltration. The correlation between the abundance of immune cells and the expression of A CTSG, B DUSP9, C CLEC10A, D CPA3, ECD1C in TCGA-SARC cohort.
