# Supplementary figures and images for: Identification and Validation of a Novel Pyroptosis-Related Gene Signature for Prognosis Prediction in Soft Tissue Sarcoma
Source: Front Genet. 2021 Dec 1;12:773373. doi: 10.3389/fgene.2021.773373 (PMC8671884; doi:10.3389/fgene.2021.773373)

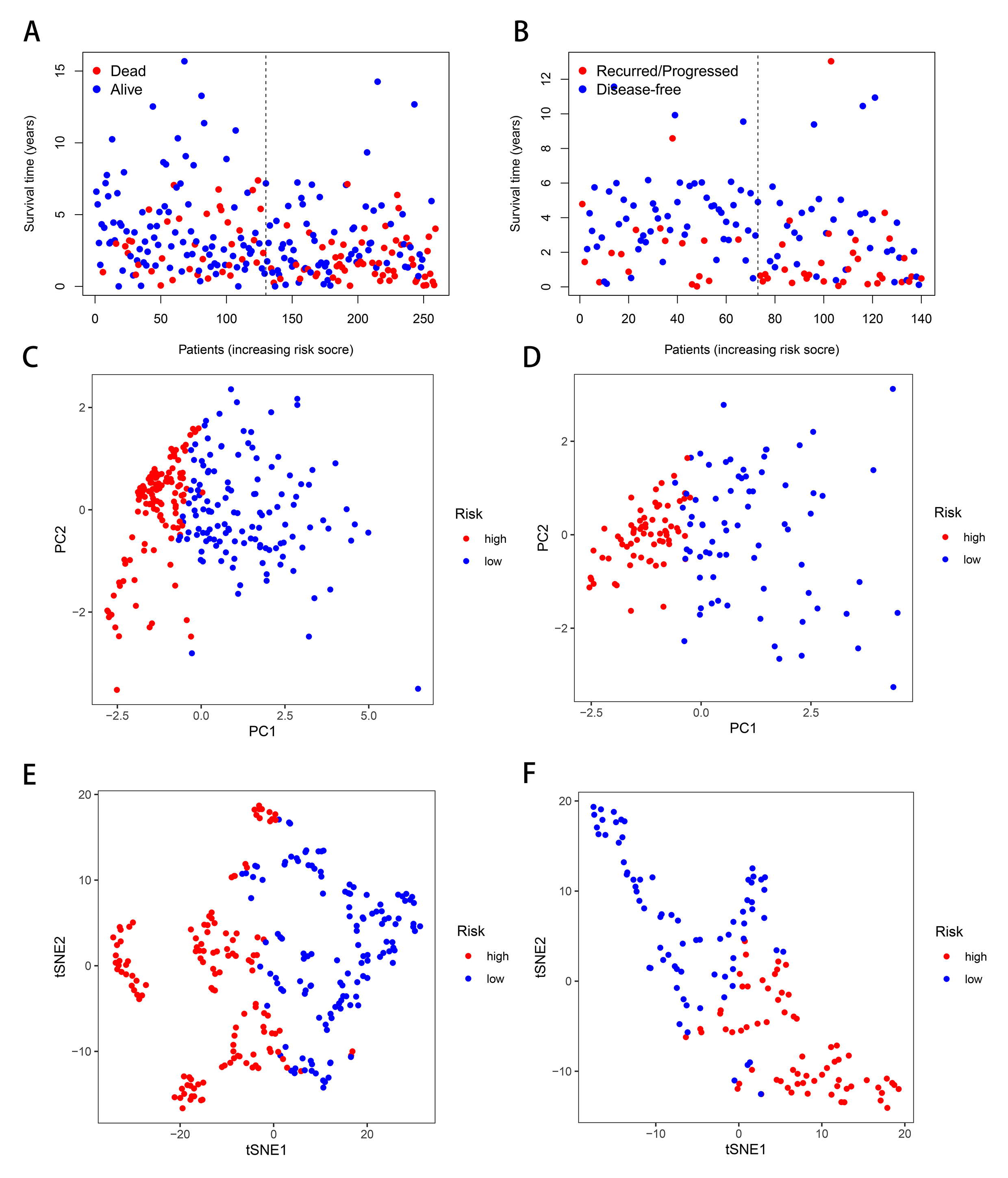

Supplement: Supplementary file 1 [file Image3.JPEG]

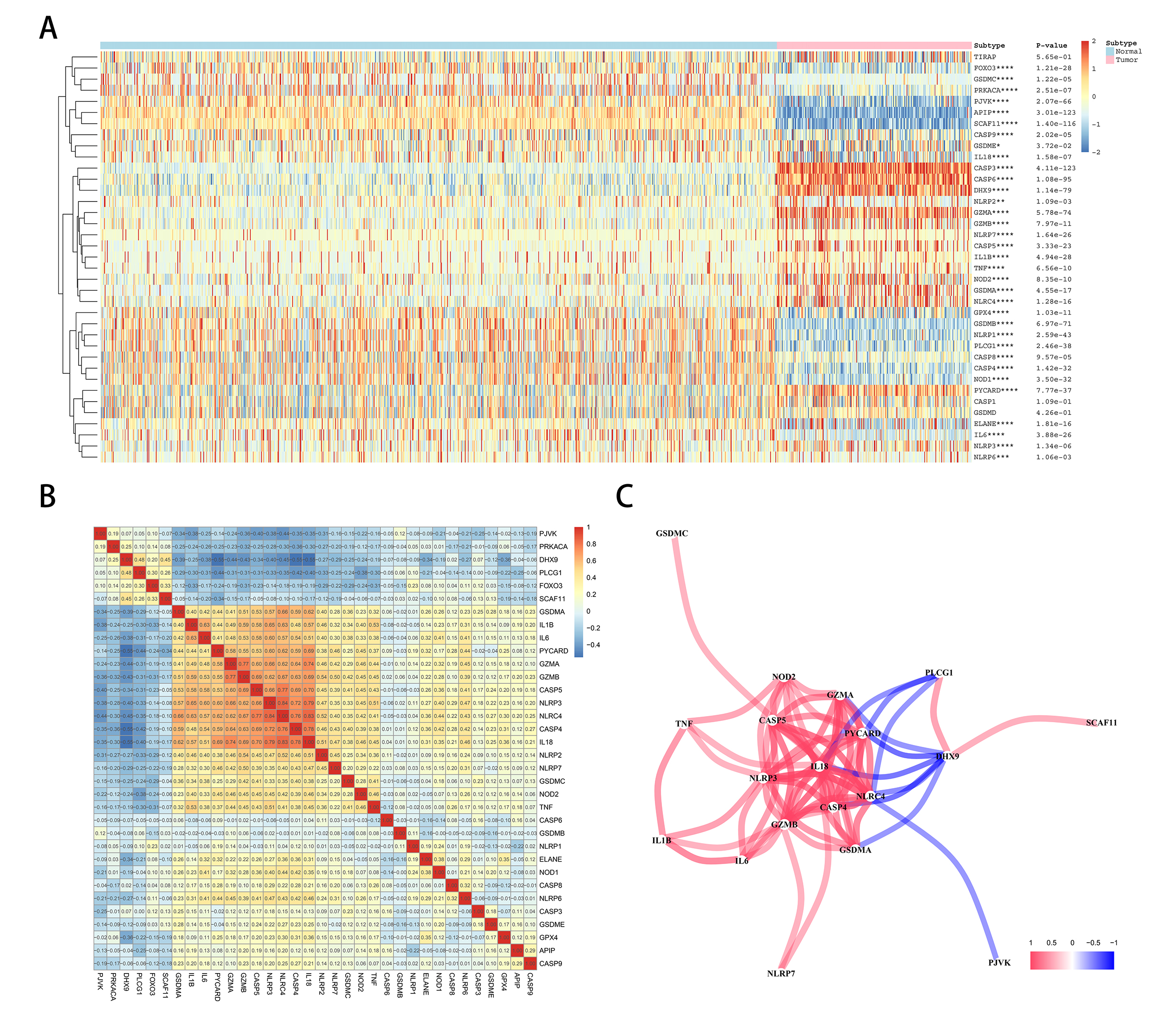

Supplement: Supplementary file 3 [file Image1.JPEG]

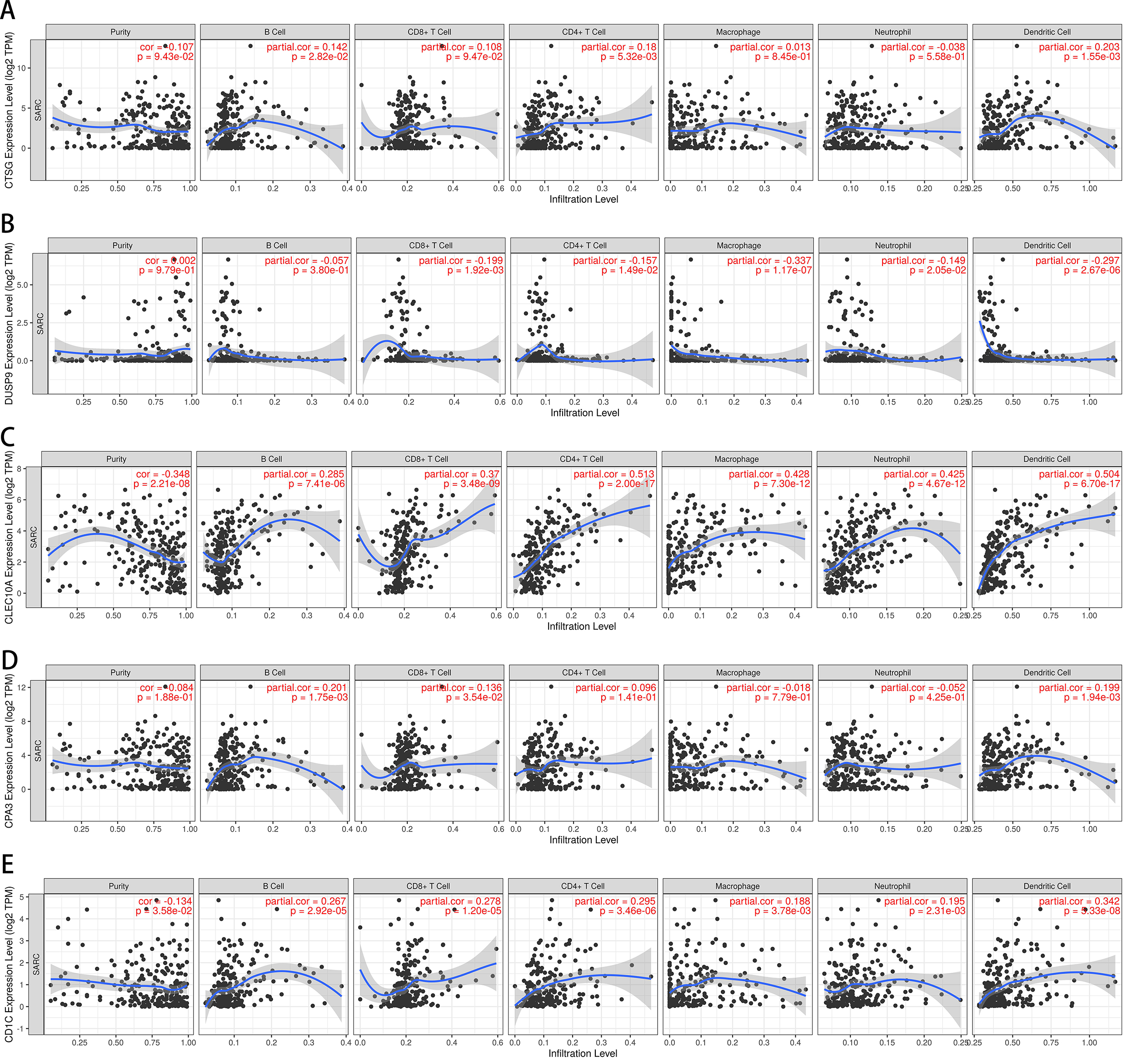

Supplement: Supplementary file 4 [file Image4.JPEG]

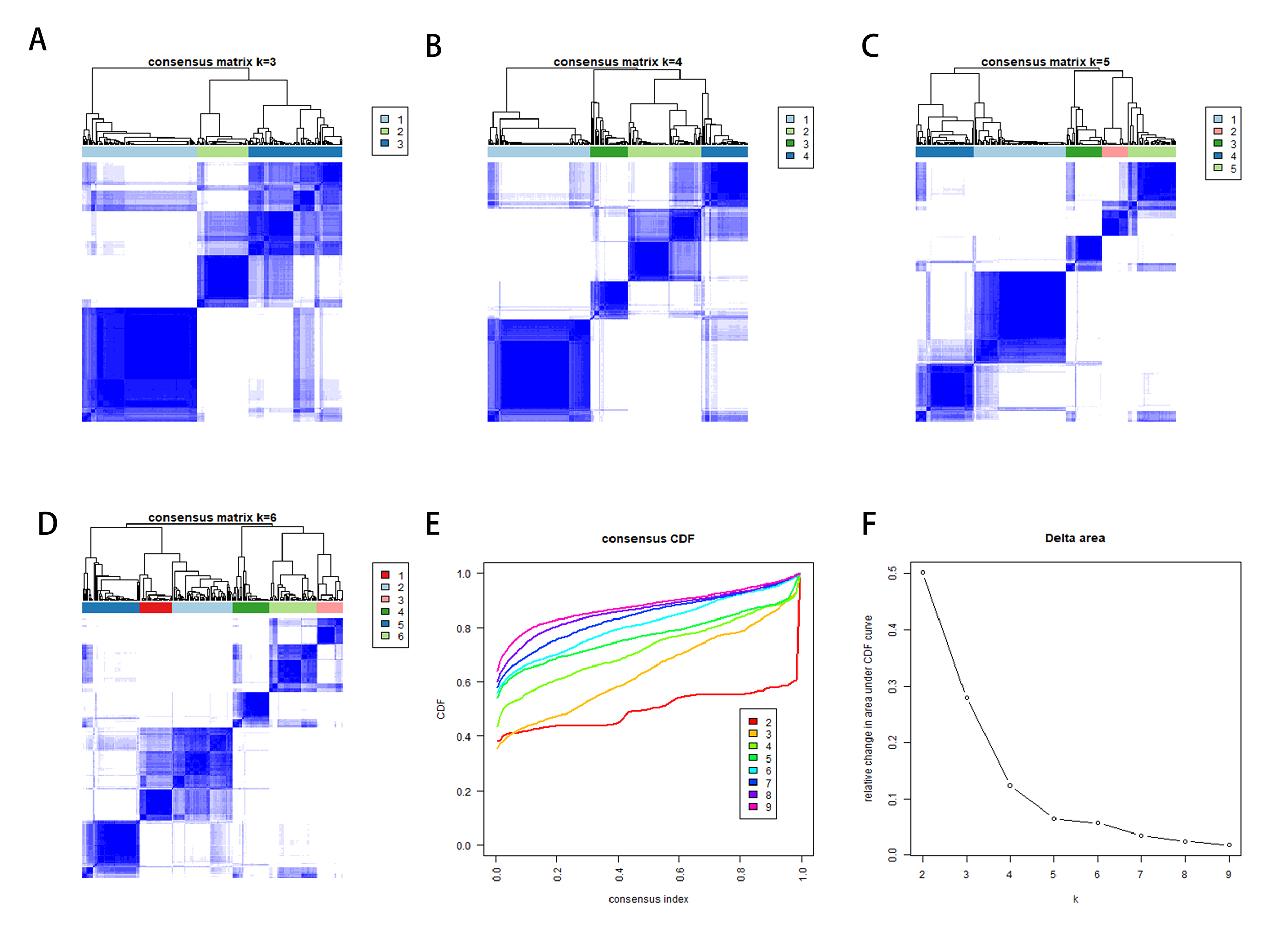

Supplement: Supplementary file 6 [file Image2.JPEG]
