# Supplementary material for: Identification and Validation of a Novel Pyroptosis-Related Gene Signature for Prognosis Prediction in Soft Tissue Sarcoma
Source: Front Genet. 2021 Dec 1;12:773373. doi: 10.3389/fgene.2021.773373 (PMC8671884; doi:10.3389/fgene.2021.773373)
Supplement: Supplementary file 2 [file Table1.DOCX]

| **Table 1. Sequences of the primers used in RT-qPCR** | |
| --- | --- |
| **Gene** | **Sequence of primer** |
| *CASP3* | F: CATGGAAGCGAATCAATGGACT |
|  | R: CTGTACCAGACCGAGATGTCA |
| *DHX9* | F: GCAGCAGAGTGTAACATCGTAG |
|  | R: ACTCAAATCGAACGCTGTAGC |
| *IL1B* | F: ATGATGGCTTATTACAGTGGCAA |
|  | R: GTCGGAGATTCGTAGCTGGA |
| *GADPH* | F: CAGGAGGCATTGCTGATGAT |
|  | R: GAAGGCTGGGGCTCATTT |
