# Supplementary material for: Identification and Validation of a Novel Pyroptosis-Related Gene Signature for Prognosis Prediction in Soft Tissue Sarcoma
Source: Front Genet. 2021 Dec 1;12:773373. doi: 10.3389/fgene.2021.773373 (PMC8671884; doi:10.3389/fgene.2021.773373)
Supplement: Supplementary file 5 [file DataSheet1.ZIP › Raw data and code/3.whole gene Exp-TCGA>Ex.docx]

The size of expression profiles of TCGA-SARC and normal human adipose and muscle in GTEx was 103 MB, which were too large to upload.

Editors and reviewers could use the UCSC Xena browser (<https://xenabrowser.net/datapages/>) to download gene expression profiles of TCGA-SARC and GTEx dataset.
