# Supplementary material for: Identification and Validation of a Novel Pyroptosis-Related Gene Signature for Prognosis Prediction in Soft Tissue Sarcoma
Source: Front Genet. 2021 Dec 1;12:773373. doi: 10.3389/fgene.2021.773373 (PMC8671884; doi:10.3389/fgene.2021.773373)
Supplement: Supplementary file 7 [file Table2.DOCX]

**Table S1 Pyroptosis-related genes in this study**

Genes Full-names Ref

APIP Apaf-1 interacting protein (Kang et al., 2014)

CASP1 cysteine-aspartic acid protease-1 (Man et al., 2017)

CASP3 cysteine-aspartic acid protease-3 (Jiang et al., 2020)

CASP4 cysteine-aspartic acid protease-4 (Man et al., 2017)

CASP5 cysteine-aspartic acid protease-5 (Man et al., 2017)

CASP6 cysteine-aspartic acid protease-6 (Zheng and Kanneganti, 2020)

CASP8 cysteine-aspartic acid protease-8 (Zheng and Kanneganti, 2020)

CASP9 cysteine-aspartic acid protease-9 (Tsuchiya et al., 2019)

DHX9 DExH-Box helicase 9 (Zhu et al., 2017)

ELANE neutrophil elastase (Kambara et al., 2018)

FOXO3 forkhead transcription factor O3 (Tajima et al., 2019)

GSDMA gasdermin A (Feng et al., 2018)

GSDMB gasdermin B (Feng et al., 2018)

GSDMC gasdermin C (Feng et al., 2018)

GSDMD gasdermin D (Feng et al., 2018)

GSDME gasdermin E (Feng et al., 2018)

GPX4 glutathione peroxidase 4 (Kang et al., 2018)

GZMA Granzyme A (Zhou et al., 2020)

GZMB Granzyme B (Zhang et al., 2020)

IL18 interleukin 18 (Man et al., 2017)

IL1B interleukin 1 beta (Man et al., 2017)

IL6 interleukin 6 (Wu et al., 2020)

NLRC4 NLR family CARD domain containing 4 (Xue et al., 2019)

NLRP1 NLR family pyrin domain containing 1 (Xue et al., 2019)

NLRP2 NLR family pyrin domain containing 1 (Xue et al., 2019)

NLRP3 NLR family pyrin domain containing 3 (Xue et al., 2019)

NLRP6 NLR family pyrin domain containing 6 (Xue et al., 2019)

NLRP7 NLR family pyrin domain containing 7 (Xue et al., 2019)

NOD1 nucleotide binding oligomerization domain containing 1 (Xue et al., 2019)

NOD2 nucleotide binding oligomerization domain containing 2 (Kang et al., 2014)

PJVK pejvakin (Feng et al., 2018)

PLCG1 phospholipase C gamma 1 (Kang et al., 2018)

PRKACA protein kinase CAMP-activated catalytic subunit Alpha (Lin et al., 2021)

PYCARD PYD and CARD domain containing (Xue et al., 2019)

SCAF11 SR-related CTD associated factor 11 (Ye et al., 2021)

TIRAP TIR domain containing adaptor protein (Bergsbaken et al., 2009)

TNF tumor necrosis factor (Shi et al., 2017)

Bergsbaken, T., Fink, S.L., and Cookson, B.T. (2009). Pyroptosis: host cell death and inflammation. *Nat Rev Microbiol* 7**,** 99-109.

Feng, S., Fox, D., and Man, S.M. (2018). Mechanisms of Gasdermin Family Members in Inflammasome Signaling and Cell Death. *J Mol Biol* 430**,** 3068-3080.

Jiang, M., Qi, L., Li, L., and Li, Y. (2020). The caspase-3/GSDME signal pathway as a switch between apoptosis and pyroptosis in cancer. *Cell Death Discov* 6**,** 112.

Kambara, H., Liu, F., Zhang, X., Liu, P., Bajrami, B., Teng, Y., Zhao, L., Zhou, S., Yu, H., Zhou, W., Silberstein, L.E., Cheng, T., Han, M., Xu, Y., and Luo, H.R. (2018). Gasdermin D Exerts Anti-inflammatory Effects by Promoting Neutrophil Death. *Cell Rep* 22**,** 2924-2936.

Kang, R., Zeng, L., Zhu, S., Xie, Y., Liu, J., Wen, Q., Cao, L., Xie, M., Ran, Q., Kroemer, G., Wang, H., Billiar, T.R., Jiang, J., and Tang, D. (2018). Lipid Peroxidation Drives Gasdermin D-Mediated Pyroptosis in Lethal Polymicrobial Sepsis. *Cell Host Microbe* 24**,** 97-108.e104.

Kang, W., Hong, S.H., Lee, H.M., Kim, N.Y., Lim, Y.C., Le Le, T.M., Lim, B., Kim, H.C., Kim, T.Y., Ashida, H., Yokota, A., Hah, S.S., Chun, K.H., Jung, Y.K., and Yang, J.K. (2014). Structural and biochemical basis for the inhibition of cell death by APIP, a methionine salvage enzyme. *Proc Natl Acad Sci U S A* 111**,** E54-61.

Lin, W., Chen, Y., Wu, B., Chen, Y., and Li, Z. (2021). Identification of the pyroptosisrelated prognostic gene signature and the associated regulation axis in lung adenocarcinoma. *Cell Death Discov* 7**,** 161.

Man, S.M., Karki, R., and Kanneganti, T.D. (2017). Molecular mechanisms and functions of pyroptosis, inflammatory caspases and inflammasomes in infectious diseases. *Immunol Rev* 277**,** 61-75.

Shi, J., Gao, W., and Shao, F. (2017). Pyroptosis: Gasdermin-Mediated Programmed Necrotic Cell Death. *Trends Biochem Sci* 42**,** 245-254.

Tajima, T., Yoshifuji, A., Matsui, A., Itoh, T., Uchiyama, K., Kanda, T., Tokuyama, H., Wakino, S., and Itoh, H. (2019). β-hydroxybutyrate attenuates renal ischemia-reperfusion injury through its anti-pyroptotic effects. *Kidney Int* 95**,** 1120-1137.

Tsuchiya, K., Nakajima, S., Hosojima, S., Thi Nguyen, D., Hattori, T., Manh Le, T., Hori, O., Mahib, M.R., Yamaguchi, Y., Miura, M., Kinoshita, T., Kushiyama, H., Sakurai, M., Shiroishi, T., and Suda, T. (2019). Caspase-1 initiates apoptosis in the absence of gasdermin D. *Nat Commun* 10**,** 2091.

Wu, X.Y., Li, K.T., Yang, H.X., Yang, B., Lu, X., Zhao, L.D., Fei, Y.Y., Chen, H., Wang, L., Li, J., Peng, L.Y., Zheng, W.J., Hou, Y., Jiang, Y., Shi, Q., Zhang, W., Zhang, F.C., Zhang, J.M., Huang, B., He, W., and Zhang, X. (2020). Complement C1q synergizes with PTX3 in promoting NLRP3 inflammasome over-activation and pyroptosis in rheumatoid arthritis. *J Autoimmun* 106**,** 102336.

Xue, Y., Enosi Tuipulotu, D., Tan, W.H., Kay, C., and Man, S.M. (2019). Emerging Activators and Regulators of Inflammasomes and Pyroptosis. *Trends Immunol* 40**,** 1035-1052.

Ye, Y., Dai, Q., and Qi, H. (2021). A novel defined pyroptosis-related gene signature for predicting the prognosis of ovarian cancer. *Cell Death Discov* 7**,** 71.

Zhang, Z., Zhang, Y., Xia, S., Kong, Q., Li, S., Liu, X., Junqueira, C., Meza-Sosa, K.F., Mok, T.M.Y., Ansara, J., Sengupta, S., Yao, Y., Wu, H., and Lieberman, J. (2020). Gasdermin E suppresses tumour growth by activating anti-tumour immunity. *Nature* 579**,** 415-420.

Zheng, M., and Kanneganti, T.D. (2020). The regulation of the ZBP1-NLRP3 inflammasome and its implications in pyroptosis, apoptosis, and necroptosis (PANoptosis). *Immunol Rev* 297**,** 26-38.

Zhou, Z., He, H., Wang, K., Shi, X., Wang, Y., Su, Y., Wang, Y., Li, D., Liu, W., Zhang, Y., Shen, L., Han, W., Shen, L., Ding, J., and Shao, F. (2020). Granzyme A from cytotoxic lymphocytes cleaves GSDMB to trigger pyroptosis in target cells. *Science* 368.

Zhu, S., Ding, S., Wang, P., Wei, Z., Pan, W., Palm, N.W., Yang, Y., Yu, H., Li, H.B., Wang, G., Lei, X., De Zoete, M.R., Zhao, J., Zheng, Y., Chen, H., Zhao, Y., Jurado, K.A., Feng, N., Shan, L., Kluger, Y., Lu, J., Abraham, C., Fikrig, E., Greenberg, H.B., and Flavell, R.A. (2017). Nlrp9b inflammasome restricts rotavirus infection in intestinal epithelial cells. *Nature* 546**,** 667-670.
