# Supplementary material for: Identification and Validation of a Novel Pyroptosis-Related Gene Signature for Prognosis Prediction in Soft Tissue Sarcoma
Source: Front Genet. 2021 Dec 1;12:773373. doi: 10.3389/fgene.2021.773373 (PMC8671884; doi:10.3389/fgene.2021.773373)
Supplement: Supplementary file 8 [file Table3.DOCX]

| **Table S2 COX regression analysis in TCGA-SARC cohort** | | | | | | | |
| --- | --- | --- | --- | --- | --- | --- | --- |
|  | **Number of Patients** | **Proportion (%)** | **Univariate COX analysis** | |  | **Multivariate COX analysis** | |
|  |  |  | **HR (95%CI)** | ***p* value** |  | **HR (95%CI)** | ***p* value** |
| **Gender** |  |  |  |  |  |  |  |
| Female | 141 | 54.40 | Reference | NA |  | Reference | NA |
| Male | 118 | 45.60 | 0.853 (0.571-1.275) | 0.437 |  | 0.824 (0.528-1.288) | 0.396 |
| **Age** |  |  |  |  |  |  |  |
| <=60 | 128 | 49.40 | Reference | NA |  | Reference | NA |
| >60 | 131 | 50.60 | 1.385 (0.929-2.065) | 0.110 |  | 1.250 (0.816-1.916) | 0.305 |
| **Race** |  |  |  |  |  |  |  |
| White | 226 | 87.30 | Reference | NA |  | Reference | NA |
| Black | 18 | 6.90 | 0.729 (0.319-1.670) | 0.455 |  | 0.544 (0.232-1.276) | 0.161 |
| Other | 15 | 5.80 | 0.786 (0.319-1.940) | 0.602 |  | 0.861 (0.334-2.220) | 0.757 |
| **Histolygy** |  |  |  |  |  |  |  |
| Leiomyosarcoma | 104 | 40.20 | Reference | NA |  | Reference | NA |
| Liposarcoma | 59 | 22.80 | 1.210 (0.735-1.991) | 0.454 |  | 1.057 (0.564-1.979) | 0.863 |
| Myxofibrosarcoma | 25 | 9.70 | 0.850 (0.413-1.750) | 0.659 |  | 1.163 (0.506-2.673) | 0.723 |
| UPS | 49 | 18.90 | 1.086 (0.608-1.939) | 0.782 |  | 1.071 (0.543-2.111) | 0.844 |
| Other | 22 | 8.50 | 0.817 (0.367-1.823) | 0.622 |  | 0.628 (0.261-1.507) | 0.297 |
| **Site** |  |  |  |  |  |  |  |
| Extremity | 84 | 32.40 | Reference | NA |  | Reference | NA |
| Other | 175 | 67.60 | 1.097 (0.716-1.679) | 0.671 |  | 1.110 (0.692-1.781) | 0.665 |
| **Tumor depth** |  |  |  |  |  |  |  |
| Deep | 185 | 71.40 | Reference | NA |  | Reference | NA |
| Superficial | 21 | 8.10 | 0.341 (0.108-1.084) | 0.068 |  | 0.441 (0.131-1.469) | 0.182 |
| Unknown | 53 | 20.50 | 1.021 (0.632-1.647) | 0.934 |  | 1.191 (0.681-2.083) | 0.540 |
| **Multifocal** |  |  |  |  |  |  |  |
| Yes | 39 | 15.10 | Reference | NA |  | Reference | NA |
| No | 198 | 76.40 | 0.980 (0.572-1.678) | 0.940 |  | 1.168 (0.652-2.093) | 0.601 |
| Unknown | 22 | 8.50 | 0.454 (0.152-1.359) | 0.158 |  | 0.475 (0.152-1.479) | 0.199 |
| **Surgical margin** |  |  |  |  |  |  |  |
| Negative | 158 | 61.00 | Reference | NA |  | Reference | NA |
| Positive | 89 | 34.40 | 2.468 (1.640-3.714) | <0.001 |  | 2.191 (1.419-3.383) | <0.001 |
| Unknown | 12 | 4.60 | 2.652 (0.987-6.844) | 0.053 |  | 2.835 (0.992-8.103) | 0.055 |
| **Postoperative radiotherapy** |  |  |  |  |  |  |  |
| Yes | 73 | 28.20 | Reference | NA |  | Reference | NA |
| No | 140 | 54.10 | 1.017 (0.637-1.625) | 0.943 |  | 1.027 (0.595-1.771) | 0.924 |
| Unknown | 46 | 17.80 | 1.114 (0.628-1.975) | 0.712 |  | 0.950 (0.469-1.926) | 0.888 |
| **Risk score** |  |  |  |  |  |  |  |
| Low | 130 | 50.20 | Reference | NA |  | Reference | NA |
| High | 129 | 49.80 | 2.336 (1.549-3.524) | <0.001 |  | 2.552 (1.636-3.979) | <0.001 |

UPS, undifferentiated pleomorphic sarcoma.
